# HPV Genotype Distribution in Cervical Intraepithelial Neoplasia among HIV-Infected Women in Pune, India

**DOI:** 10.1371/journal.pone.0038731

**Published:** 2012-06-19

**Authors:** Arati Mane, Amit Nirmalkar, Arun R. Risbud, Sten H. Vermund, Sanjay M. Mehendale, Vikrant V. Sahasrabuddhe

**Affiliations:** 1 National AIDS Research Institute, Pune, India; 2 Vanderbilt Institute for Global Health, Vanderbilt University School of Medicine, Nashville, Tennessee, United States of America; 3 National Institute of Epidemiology, Chennai, India; National Cancer Institute, United States of America

## Abstract

**Background:**

The distribution of HPV genotypes, their association with rigorously confirmed cervical precancer endpoints, and factors associated with HPV infection have not been previously documented among HIV-infected women in India. We conducted an observational study to expand this evidence base in this population at high risk of cervical cancer.

**Methods:**

HIV-infected women (N = 278) in Pune, India underwent HPV genotyping by Linear Array assay. Cervical intraepithelial neoplasia (CIN) disease ascertainment was maximized by detailed assessment using cytology, colposcopy, and histopathology and a composite endpoint.

**Results:**

CIN2+ was detected in 11.2% while CIN3 was present in 4.7% participants. HPV genotypes were present in 52.5% (146/278) and ‘carcinogenic’ HPV genotypes were present in 35.3% (98/278) HIV-infected women. ‘Possibly carcinogenic’ and ‘non/unknown carcinogenic’ HPV genotypes were present in 14.7% and 29.5% participants respectively. Multiple (≥2) HPV genotypes were present in half (50.7%) of women with HPV, while multiple ‘carcinogenic’ HPV genotypes were present in just over a quarter (27.8%) of women with ‘carcinogenic’ HPV. HPV16 was the commonest genotype, present in 12% overall, as well as in 47% and 50% in CIN2+ and CIN3 lesions with a single carcinogenic HPV infection, respectively. The carcinogenic HPV genotypes in declining order of prevalence overall included HPV 16, 56, 18, 39, 35, 51, 31, 59, 33, 58, 68, 45 and 52. Factors independently associated with ‘carcinogenic’ HPV type detection were reporting ≥2 lifetime sexual partners and having lower CD4+ count. HPV16 detection was associated with lower CD4+ cell counts and currently receiving combination antiretroviral therapy.

**Conclusion:**

HPV16 was the most common HPV genotype, although a wide diversity and high multiplicity of HPV genotypes was observed. Type-specific attribution of carcinogenic HPV genotypes in CIN3 lesions in HIV-infected women, and etiologic significance of concurrently present non/unknown carcinogenic HPV genotypes await larger studies.

## Introduction

Human immunodeficiency virus (HIV)-infected women in India and other developing countries are living longer in recent years as a result of improved access to affordable combination antiretroviral therapy (cART) drugs. Yet, access to services for prevention of common HIV-associated malignancies such as invasive cervical cancer (ICC) caused by carcinogenic human papillomavirus (HPV) remains inadequate. Thus, HIV-infected women remain at increased risk for HPV infection and cervical precancerous lesions (i.e., cervical intraepithelial neoplasia [CIN]) progressing to ICC. [1], [2], [3].

Very few studies have described HPV genotype composition among HIV-infected women in India. [4], [5], [6] None of these studies have correlated genotype-specific HPV prevalence against rigorously verified cervical disease endpoints and immune status of HIV-infected women. The development of this evidence is critical to informing the design and delivery of HPV vaccination as well as HPV-based screening strategies for HIV-infected women.

We conducted an observational study among HIV-infected women in Pune, India to expand the evidence base of HPV genotype distribution in this population.

## Methods

### Ethics Statement

The study protocol was approved by the institutional ethical review boards of the National AIDS Research Institute, Pune, India and Vanderbilt University, Nashville, USA. All participants gave written, informed consent.

### Study Setting and Participants

The study was carried out in an outpatient gynecology clinic in a tertiary care hospital in Pune, India as part of the NIH-ICMR funded India-US HIV-Cervical Cancer Prevention Research Consortium. Consecutive women with documented serologic evidence of HIV infection were enrolled in the study. Participants were recruited regardless of their CD4+ cell counts or current status of receiving cART drugs. Exclusion criteria included a positive urine pregnancy test, debilitating illness that may preclude a pelvic examination, prior history of screening or treatment for cervical neoplasia, prior hysterectomy, and presence of current sexually transmitted infection.

### Study Procedures

After explanation of study procedures and written informed consent, a structured questionnaire was administered to interview the participants and collect their sociodemographic information as well as key bio-behavioral risk factors relevant to HIV/AIDS and cervical cancer. Blood samples were obtained for CD4+ T-cell counts estimation [FACSCount™ flow cytometry, Becton, Dickinson and Company, Franklin Lakes, NJ, USA]. All enrolled women underwent a complete physical, pelvic, and colposcopic examination. Trained nurses collected cervical samples by spatula and cytobrush for conventional cervical cytology and HPV testing/genotyping. A standardized non-invasive colposcopy examination was performed on all participants by trained gynecologists. Confirmatory procedures for histology [by cervical punch biopsy, endocervical curettage (ECC), and loop electrosurgical excision procedures (LEEP)] were advised and performed only on consenting participants with clinical evidence of cervical abnormalities.

Cervical cytology and histopathology samples were analyzed by two experienced pathologists who reported diagnosis by consensus. The pathologists did not have knowledge of the HPV status of the participant. Cervical cytology results were reported as per revised (2001) Bethesda classification. [7] Colposcopy and histology results were reported as per the Richart CIN system. [8] We used results of both colposcopy/histology and cytology results to define distinct disease stages of increasing severity of CIN disease in the following categories: No CIN (normal colposcopy/histology *and* normal cytology), CIN1 (CIN1 lesions on colposcopy/histology *or* cervical cytology results of either atypical squamous cells of undetermined significance [ASC-US] or low-grade squamous intraepithelial cells [LSIL]), CIN2 (CIN2 lesions on colposcopy/histology *or* high-grade squamous intraepithelial cells [HSIL] on cervical cytology) and CIN3 (CIN3 on colposcopy/histology). This classification ensured that the most severe (abnormal) cellular or tissue detection of dysplastic changes were included in the appropriate CIN disease status category.

### HPV Genotyping

We performed HPV genotyping on cervical specimens using PCR-based amplification of target DNA using the Linear Array® HPV genotyping test (LA-HPV) (Roche Molecular Systems, Pleasanton, CA, USA), an enhanced and commercialized version of the PGMY line blot assay (PGMY-LB) [9], [10], [11] The pool of consensus L1 PGMY09/11 primers used in this assay is designed to amplify HPV-DNA from 37 genotypes. These include genotypes characterized by WHO/IARC as ‘carcinogenic’ (13 genotypes): HPV16, 18, 31, 33, 35, 39, 45, 51, 52, 56, 58, 59, 68, ‘possibly carcinogenic’ (7 genotypes): HPV26, 53, 66, 67, 70, 73, 82 and ‘non carcinogenic/unknown carcinogenicity’ (17 genotypes): HPV6, 11, 40, 42, 54, 55, 61, 62, 64, 69, 71, 72, 81, 83, 84, CP6108, IS39. [12] DNA was extracted from specimen aliquot by AmpliLute liquid medium extraction kit (Roche Molecular Diagnostics, Branchburg, NJ, USA). The PCR amplicons were denatured and subjected to hybridization on LA HPV genotyping strips coated with HPV type-specific and human β-globin probes according to manufacturer’s instructions. The biotin-labeled amplicons hybridized to the probes only if the type-specific sequence matched those of the amplicons. The biotin-labeled amplicons were detected by colorimetric development and the results were read visually by comparing the pattern of colored lines to the provided reference guide. Each run was performed with negative and positive controls provided by the manufacturer to monitor the quality and performance of the assay.

### Statistical Analysis

Data were analyzed using STATA intercooled version 10.0 and IBM SPSS Statistics 19. We analyzed prevalence of individual HPV genotypes (classified by carcinogenic risk categories and number of HPV genotypes per woman) in age categories, CD4+ cell strata, and cervical disease stages (No CIN, CIN1, CIN2, and CIN3). Chi-square test for trend was used to analyze trends in proportion of women with prevalent HPV genotypes and CIN status.

We fit bivariate and multivariable logistic regression models to identify associations between sociodemographic characteristics (age, marital status, education, family income) and bio-behavioral factors (parity, age at first sex, number of lifetime sexual partners, history of STI, tobacco use, CD4+ cell counts, and ART status) with HPV infection status. The dependent variables included (i) any prevalent HPV genotype, (ii) any prevalent ‘carcinogenic’ HPV genotypes, (iii) single carcinogenic HPV genotype, (iv) multiple carcinogenic HPV genotype, (v) HPV16 (the most common/most carcinogenic genotype), and (vi) any non-HPV16 carcinogenic type. We also evaluated the risk (approximated by the prevalence odds ratios and their 95% confidence intervals) of having high-grade cervical precancerous lesions (CIN3 and CIN2+) with the presence of individual carcinogenic HPV genotypes (any, single, and multiple), with adjustment (as appropriate) for age, number of lifetime sexual partners, CD4+ cell counts, and presence of other carcinogenic HPV types. In an exploratory analysis, this risk of CIN3 and CIN2+ lesions was also estimated for combinations of individual carcinogenic HPV genotypes (single and multiple) with the concurrent presence of single and multiple non-carcinogenic types.

## Results

### Population Characteristics

A total of 278 HIV-infected women were enrolled for this study. The mean age was 32.3 years (S.D.: ±5.3), a third (89/278, 32%) were married and cohabiting with their husband, a third (92/278, 33.1%) were illiterate, and a majority (84/278, 57.9%) reported their family income of <2500 Indian Rupees per month (approximately US$55 at the time of the study). Half (137/278, 49.2%) of the participants reported age of first sexual intercourse as <18 years, while about one fifth (51/278, 18.3%) reported to have ≥2 lifetime sexual partners. Mean and median CD4+ cell counts were 411 /µL (S.D. ±214) and 372 /µL (interquartile range: 241–556) respectively.

### Cervical Disease Status on Cytology and Colposcopy-histopathology

Cytology results revealed 7 women with HSIL, 41 with LSIL, 47 with ASC-US, 165 women with no squamous cell abnormality on cytology, while 18 women had inadequately stained smears. Colposcopic-histopathologic diagnoses revealed 13 women with CIN3, 16 women with CIN2, 46 women with CIN1, 193 women without any CIN abnormality, while colposcopy was unsatisfactory in 10 women. Three women had both unsatisfactory colposcopy and also cervical smears that were inadequately stained, such that the final composite cytologic-colposcopic-histopathological CIN disease status was able to be determined for 275 out of 278 women.

The composite cytology-colposcopic-histopathological CIN diagnosis (N = 275) thus included no evidence of CIN in 143/275 (52%) women, CIN1 (CIN1/ASC-US/LSIL) in 101/275 (36.7%), CIN2 (CIN2/HSIL) in 18/275 (6.5%), and CIN3 in 13/275 (4.7%) women. Thus, the prevalence of CIN2+ was 11.2% (31/275) and that of CIN3 was 4.7% (13/275) in this population.

### HPV Genotypes by Carcinogenicity Grouping

At least one HPV genotype was detected in 146/278 (52.5%) participants, while multiple (≥2) HPV genotypes were present in 74/146 (50.7%) women. The number of HPV genotypes per woman ranged between 0–8, with a median of 1 genotype per woman. ‘Carcinogenic’ HPV genotypes were present in 98/278 (35.3%) women, ‘possibly carcinogenic’ genotypes in 41/278 (14.7%) while ‘non/unknown carcinogenic’ types were detected in 82/278 (29.5%) women. Out of 98 women with presence of any ‘carcinogenic’ HPV genotypes, 71/98 (72.4%) had a single ‘carcinogenic’ HPV genotype, 23/98 (23.4%) had two ‘carcinogenic’ HPV genotypes, while 4/98 (4.1%) had three ‘carcinogenic’ HPV genotypes, thus a total of 129 individual carcinogenic HPV infections were identified. Only 17/278 (6.1%) women with ‘possibly carcinogenic’ HPV genotypes and only 35/278 (12.6%) women with ‘non/unknown carcinogenic’ HPV genotypes were present without the concurrent presence of ‘carcinogenic’ HPV genotypes.

**Figure 1:**
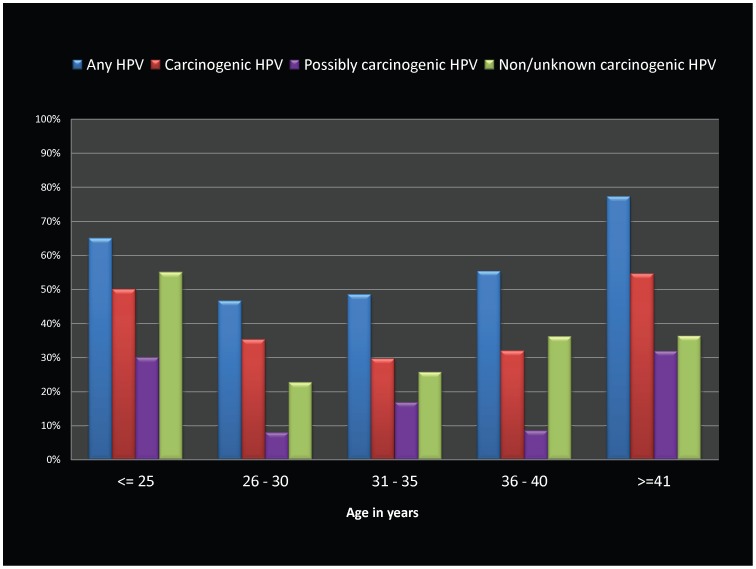
Bar graphs showing HPV genotype prevalence by age categories among HIV-infected women in Pune, India. HPV prevalence levels (as percentages) are displayed on the Y-axis, with various carcinogenicity groupings (any HPV type, carcinogenic HPV type, possibly carcinogenic HPV types, and non/unknown carcinogenic types) shown as individual bar graphs grouped by age categories (≤25, 26–30, 31–35, 36–40, & ≥41 years) on X-axis.

**Figure 2:**
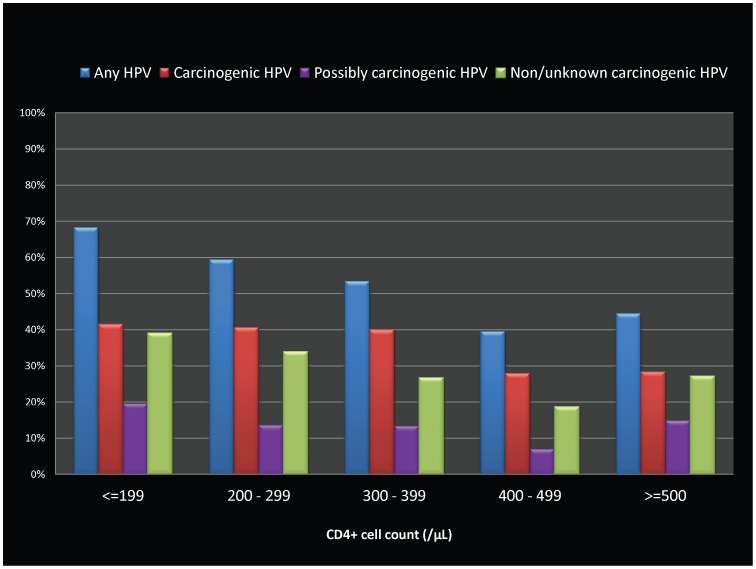
Bar graphs showing HPV genotype prevalence by CD4+ cell count categories among HIV-infected women in Pune, India. HPV prevalence levels (as percentages) are displayed on the Y-axis, with various carcinogenicity groupings (any HPV type, carcinogenic HPV type, possibly carcinogenic HPV types, and non/unknown carcinogenic types) shown as individual bar graphs grouped by CD4+ count categories (≤199, 200–299, 300–399, 400–499, ≥500 /µL) on X-axis.

The relative proportions of HPV infections (by carcinogenic risk categories) by age and CD4+ cell count categories are shown in Figures 1 and 2. The age-specific prevalence revealed a mixed pattern by carcinogenicity grouping, reflecting a >50% prevalence in both the youngest (≤25 years of age) as well as the oldest (≥41 years) age categories in the study population. (Figure 1) ‘Carcinogenic’ HPV genotypes were higher in prevalence than the other categories regardless of CD4+ counts, although the prevalence of HPV in all carcinogenic categories was uniformly lower than 30% with CD4+ cell counts ≥400 /µL. (Figure 2).

### Relative Prevalence of HPV Genotypes

Of the 37 HPV genotypes identifiable by the Linear Array assay, all except one (HPV69) were detected in the 278 samples. The commonest 10 genotypes were HPV16 (12.2%), HPV62 (7.3%), HPV71 (6.5%), HPV53 (6.2%), HPV42 (5.8%), HPV56 and HPV66 (both 4.4%), HPV18 and HPV39 (both 4%), and HPV35 and HPV51 (both 3.6%). (Table 1) The carcinogenic HPV types in declining order of prevalence included HPV 16, 56, 18, 39, 35, 51, 31, 59, 33, 58, 68, 45 and 52. (Table 1).

**Table 1:** Prevalence of HPV genotypes, overall and stratified by CIN status, among HIV-infected women in Pune, India.

	Overall (N = 278)		Cervical neoplasia disease status (N = 275)				
TOTAL			No CIN = 143	CIN1 = 101	CIN2 = 18	CIN3 = 13	p-value for trend
	N	%, (95% CI)	%, (95% CI)	%, (95% CI)	%, (95% CI)	%, (95% CI)	
Any HPV	146	52.5% (46.6–58.4)	37.1% (31.4–42.8)	66.3% (60.7–71.9)	72.2% (66.9–77.5)	92.3% (89.2–95.5)	<0.001
Carcinogenic HPV	98	35.3% (29.7–40.9)	21.7% (16.8–26.6)	43.6% (37.7–49.5)	66.7% (61.1–72.3)	76.9% (71.9–81.9)	<0.001
Possibly Carc. HPV	41	14.9% (10.7–19.0)	8.4%(5.1–11.7)	23.8% (18.8–28.8)	5.6% (2.9–8.3)	30.8% (25.3–36.3)	0.01
Non/Un. Carc. HPV	82	29.8% (24.4–35.2)	22.4% (17.5–27.3)	36.6% (30.9–42.3)	50% (44.1–55.9)	30.8% (25.3–36.3)	0.02
Single Carc. HPV	70	25.5% (20.4–30.6)	18.8% (14.2–23.4)	33.7% (28.1–39.3)	53.9% (48–59.8)	72.7% (67.4–77.9)	<0.001
Multiple Carc. HPV	27	9.8% (6.3–13.3)	4.3% (1.9–6.7)	20.8% (16–25.6)	45.5% (39.2–51.4)	40.0% (34.2–45.8)	<0.001
**Carcinogenic HPV genotypes**							
HPV 16	34	12.2% (8.4–16.0)	5.6% (2.9–8.3)	14.9% (10.7–19.1)	27.8% (22.5–33.1)	38.5% (32.6–44.3)	<0.001
HPV 18	11	4.0% (1.7–6.3)	2.1% (0.4–3.8)	5.0% (2.4–7.6)	11.1% (7.4–14.8)	7.7% (4.6–10.9)	0.06
HPV 31	9	3.2% (1.1–5.3)	0.7% (0–1.7)	5.9% (3.1–8.7)	5.6% (2.9–8.3)	7.7% (4.6–10.9)	0.03
HPV 33	7	2.5% (0.7–4.3)	0.7% (0–1.7)	3.0% (0.9–5.0)	5.6% (2.9–8.3)	15.4% (11.1–19.7)	<0.005
HPV 35	10	3.6% (1.4–5.8)	2.8% (0.9–4.8)	4.0% (1.7–6.3)	11.1% (7.4–14.8)	0%	0.52
HPV 39	11	4.0% (1.7–6.3)	2.8% (0.9–4.8)	5.0% (2.4–7.6)	5.6% (2.9–8.3)	7.7% (4.6–10.9)	0.26
HPV 45	3	1.1% (0–2.3)	0%	2.0% (0.4–3.7)	5.6% (2.9–8.3)	0%	0.13
HPV 51	10	3.6% (1.4–5.8)	2.1% (0.4–3.8)	5.0% (2.4–7.6)	11.1% (7.4–14.8)	0%	0.30
HPV 52	3	1.1% (–0.1–2.3)	1.4% (0.01–2.8)	1.0% (–0.2–2.9)	0%	0%	0.51
HPV 56	12	4.3% (1.9–6.7)	2.8% (0.9–4.8)	5.0% (2.4–7.6)	16.7% (12.3–21.1)	0%	0.22
HPV 58	6	2.2% (0.5–3.9)	1.4% (0.01–2.8)	2.0% (0.4–3.7)	5.6% (2.9–8.3)	7.7% (4.6–10.9)	0.11
HPV 59	8	2.9% (0.9–4.9)	2.8% (0.9–4.8)	3.0% (0.9–5.0)	5.6% (2.9–8.3)	0%	0.96
HPV 68	5	1.8% (0.2–3.4)	0.7% (0–1.7)	3.0% (0.9–5.0)	0%	7.7% (4.6–10.9)	0.12
**‘Possibly’ carcinogenic HPV genotypes**							
HPV 26	2	0.7% (0–1.7)	1.4% (0.01–2.8)	0%	0%	0%	0.26
HPV 53	17	6.1% (3.3–8.9)	3.5% (1.3–5.7)	9.9% (6.4–13.4)	0%	15.4% (11.1–19.7)	0.11
HPV 66	12	4.3% (1.9–6.7)	1.4% (0.01–2.8)	8.9% (5.5–12.3)	5.6% (2.9–8.3)	0%	0.22
HPV 67	2	0.7% (0–1.7)	0%	1.0% (0–2.2)	0%	7.7% (4.6–10.9)	0.02
HPV 70	6	2.2% (0.5–3.9)	2.1% (0.4–3.8)	3.0% (0.9–5.0)	0%	0%	0.67
HPV 73	5	1.8% (0.2–3.4)	0%	4.0% (1.7–6.3)	0%	7.7% (4.6–10.9)	0.03
HPV 82	2	0.7% (0–1.7)	0.7% (0–1.7)	1.0% (0–2.2)	0%	0%	0.81
**Individual ‘Non-carcinogenic’ or ‘Unknown carcinogenic’ HPV genotype**							
HPV 6	2	0.7% (0–1.7)	0.7% (0–1.7)	0%	5.6% (2.9–8.3)	0%	0.53
HPV 11	1	0.4% (0–1.1)	0%	1.0% (0–2.2)	0%	0%	0.65
HPV 40	3	1.1% (0–2.3)	0.7% (0–1.7)	2.0% (0.4–3.7)	0%	0%	0.95
HPV 42	16	5.8% (3.0–8.6)	4.9% (2.4–7.5)	6.9% (3.9–9.9)	5.6% (2.9–8.3)	7.7% (4.6–10.9)	0.57
HPV 54	6	2.2% (0.5–3.9)	1.4% (0.01–2.8)	3.0% (0.9–5.0)	5.6% (2.9–8.3)	0%	0.55
HPV 55	1	0.4% (0–1.1)	0%	1% (0–2.2)	0%	0%	0.65
HPV 61	9	3.2% (1.1–5.3)	2.8% (0.9–4.8)	5.0% (2.4–7.6)	0%	0%	0.75
HPV 62	20	7.2% (4.2–10.2)	5.6% (2.9–8.3)	8.9% (5.5–12.3)	11.1% (7.4–14.8)	7.7% (4.6–10.9)	0.36
HPV 64	2	0.7% (0–1.7)	0%	2.0% (0.4–3.7)	0%	0%	0.53
HPV 69	0	–	–	–	–	–	–
HPV 71	18	6.5% (3.6–9.4)	6.3% (3.4–9.2)	6.9% (3.9–9.9)	11.1% (7.4–14.8)	0%	0.88
HPV 72	13	4.7% (2.2–7.2)	2.1% (0.4–3.8)	5.9% (3.1–8.7)	16.7% (12.3–21.1)	7.7% (4.6–10.9)	0.02
HPV 81	6	2.2% (0.5–3.9)	0%	4.0% (1.7–6.3)	5.6% (2.9–8.3)	7.7% (4.6–10.9)	0.01
HPV 83	3	1.1% (0–2.3)	1.4% (0.01–2.8)	1.0% (0–2.2)	0%	0%	0.51
HPV 84	8	2.9% (0.9–4.9)	2.1% (0.4–3.8)	5.0% (2.4–7.6)	0%	0%	0.96
HPV CP6108	5	1.8% (0.2–3.4)	1.4% (0.01–2.8)	2.0% (0.4–3.7)	5.6% (2.9–8.3)	0%	0.65
HPV IS39	1	0.4% (0–1.1)	0%	1% (0–2.2)	0%	0%	0.65

Footnotes to Table 1: Abbreviations: ‘Carc’: Carcinogenic, ‘Non/Un.’: Non/unknown, CIN: cervical intraepithelial neoplasia, HPV: human papillomavirus, 95%CI: Lower limits and upper limits of the 95% Confidence intervals.

* CIN1 =  CIN1 on Colposcopy/histopathology & ASC-US/LSIL on cytology, CIN2 =  CIN2 on colposcopy/histopathology & HSIL on cytology; 3 women did not undergo colposcopy or cytology, hence the sum of numbers of women with confirmed cervical disease status is n = 275, not n = 278.

Among the 98 women with a total of 129 ‘carcinogenic’ HPV infections, HPV16 was the most common (34/129, 26%), although all 12 other ‘carcinogenic’ HPV genotypes were also present. (data not shown) Among women with presence of any single ‘carcinogenic’ HPV infection, as well as in women with evidence of CIN2+ and CIN3 lesions with single carcinogenic infections, HPV16 was still the commonest genotype, with an increasing proportion of 34%, 47%, and 50% respectively. Other ‘carcinogenic’ genotypes-HPV33, HPV39, HPV31, HPV56 and HPV35 were also present (in respectively decreasing fractions) in women with CIN2+ and CIN3 lesions with single carcinogenic HPV genotypes.

At least one HPV genotype was present in 37.1% women with no CIN lesions, 66.3% in CIN1, 72.2% in CIN2 and 92.3% in CIN3 (p-for trend <0.001). (Table 1) The trend for increasing prevalence of HPV genotypes with increasing severity of cervical disease was also significant for any ‘carcinogenic’ HPV genotype (p<0.001), any ‘possibly carcinogenic’ type (p = 0.01), any ‘non/unknown carcinogenic’ HPV genotype (p = 0.02), any single or multiple ‘carcinogenic’ HPV genotype (p<0.001 for both) as well as for HPV16 (P<0.001), HPV31 (p = 0.03), HPV33 (p<0.005) among ‘carcinogenic’ HPV genotypes. (Table 1).

### Risk Factors for HPV Type Positivity

Table 2 shows the results of adjusted (multivariable) models of factors associated with positivity by any HPV genotype, ‘carcinogenic’ HPV genotypes (any, single, and multiple) as well as HPV16 and non-HPV16 carcinogenic types. The significant factors associated with detection of any HPV genotype reporting ≥2 lifetime sexual partners (Adjusted OR [AOR] 2.72, 95%CI 1.33–5.56), and having a lower CD4+ counts (AOR = 1.21, 95%CI = 1.06–1.37, with each 100 units/µL decline). Among the significant factors associated with any ‘carcinogenic’ HPV genotype detection were reporting ≥2 lifetime sexual partners (AOR 2.41, 95%CI 1.27–4.58) and having a lower CD4+ count (AOR 1.18, 95%CI 1.03–1.33, with each 100 units/µL decline). Whereas ≥2 lifetime number of sexual partners and lower CD4+ count were also significant risk factors for having any multiple carcinogenic HPV infections, none of these (or any other ) factors were significantly associated with presence of single carcinogenic HPV infection. Lower CD4+ cell counts (AOR 1.35, 95%CI 1.09–1.67) and currently being on cART (AOR 3.47, 95%CI 1.40–8.59) were both statistically significant factors associated with presence of HPV16, whereas these were not associated with presence of non-HPV16 carcinogenic types.

**Table 2:** Association between participants’ characteristics and presence of HPV (any HPV types, carcinogenic HPV types, single carcinogenic HPV type, multiple carcinogenic HPV types, HPV16, and non-HPV16 carcinogenic types) in HIV-infected women in Pune, India: results of multivariable logistic regression analyses.

	N	Any HPV type	Any Carcinogenic HPV type	Any Single Carcinogenic HPV type	Multiple Carcinogenic HPV types	HPV16	Non-HPV16 Carcinogenic HPV types
		*AOR [95%CI]*	*AOR [95%CI]*	*AOR [95%CI]*	*AOR [95%CI]*	*AOR [95%CI]*	*AOR [95%CI]*
**Age** (per year increase)	278	1.04 [0.98–1.09]	1.01 [0.96–1.07]	1.04 [0.98–1.10]	0.92 [0.82–1.03]	0.98 [0.90–1.06]	1.03 [0.96–1.09]
**Marital Status**							
Married, cohabiting	89	0.7 [0.38–1.30]	0.88 [0.47–1.63]	0.66 [0.33–1.34]	2.02 [0.68–6.02]	0.88 [0.36–2.15]	0.89 [0.43–1.83]
Non-cohabiting/others	189	1	1	1	1	1	1
**Education**							
Primary school or less	92	1.44 [0.76–2.73]	1.25 [0.65–2.40]	1.11 [0.53–2.31]	1.62 [0.50–5.25]	2.05 [0.80–5.25]	1.0 [0.45–2.24]
High school and above	186	1	1	1	1	1	1
**Family income (Rs)**							
<2500 per month	161	0.60 [0.33–1.09]	0.76 [0.42–1.38]	0.84 [0.43–1.64]	0.47 [0.16–1.40]	0.62 [0.26–1.47]	0.85 [0.43–1.71]
> = 2500 per month	116	1	1	1	1	1	1
**Parity**							
4 or more	76	1.35 [0.74–2.49]	1.11 [0.60–2.05]	1.16 [0.59–2.29]	0.73 **[**0.23–2.34**]**	1.26 [0.51–3.10]	1.0 [0.49–2.06]
3 or less	202	1	1	1	1	1	1
**Age at first sex**							
< = 18 years	137	1.66 [0.94–2.96]	1.16 [0.65–2.09]	1.30 [0.68–2.48]	0.80 [0.28–2.33]	1.08 [0.46–2.54]	1.13 [0.57–2.26]
>18 years	141	1	1	1	1	1	1
**Lifetime sex partners**							
2 or more	49	**2.72 [1.33–5.56]**	**2.25 [1.16–4.37]**	1.79 [0.86–3.73]	**3.95 [1.28–12.26]**	1.07 [0.39–2.97]	**2.58 [1.24–5.39]**
One	227	1	1	1	1	1	1
**Past history of STI**							
Yes	89	0.83 [0.46–1.47]	0.93 [0.51–1.68]	0.88 [0.46–1.69]	1.16 [0.38–3.55]	1.12 [0.47–2.66]	0.86 [0.43–1.72]
No	188	1	1	1	1	1	1
**Tobacco use**							
Yes	76	1.28 [0.70–2.36]	1.26 [0.69–2.29]	1.32 [0.69–2.54]	0.89 [0.29–2.69]	1.04 [0.43–2.51]	1.31 [0.66–2.61]
No	202	1	1	1	1	1	1
**CD4+** (decline by 100)	269	**1.21 [1.06–1.37]**	**1.18 [1.04–1.35]**	1.09 [0.99–1.25]	**1.59 [1.19–2.13]**	**1.35 [1.09–1.67]**	1.09 [0.94–1.26]
**Currently on ART**							
Yes	154	1.41 [0.82–2.43]	1.46 [0.84–2.55]	1.49 [0.80–2.76]	1.23 [0.44–3.41]	**3.47 [1.40–8.59]**	0.96 [0.51–1.81]
No	123	1	1	1	1	1	1

Footnotes to Table 2: Abbreviations: HPV: human papillomavirus, STI: sexually transmitted infection, ART: antiretroviral therapy,

AOR: adjusted odds ratio, 95%CI: Lower limits and upper limits of the 95% Confidence intervals. All Odds ratios presented in this table are adjusted (through multivariable logistic regression) for the covariates presented.

### Association of Carcinogenic HPV Genotypes with High Grade CIN Lesions

When dichotomized at the high grade cervical neoplasia thresholds (CIN2+ and CIN3) thresholds of the cervical disease status, women with any HPV infection had higher risk for CIN2+ [Odds Ratio (OR) 4.31(95%CI: 1.71–10.87)] and CIN3 lesions [OR 11.64 (95%CI:1.49–90.80)] than women without HPV infection. Similarly, women with any ‘carcinogenic’ HPV genotypes had higher risk for CIN2+ [OR 5.51 (95%CI: 2.42–12.53] and CIN3 lesions [OR 6.71(95%CI: 1.80–24.99] than women without ‘carcinogenic’ HPV infection. Individually, HPV16 was associated with higher risk of being present in CIN3 (vs. ≤CIN2) and CIN2+ (vs. ≤CIN1) lesions (Odds ratios [OR] 5.2 and 6.7, respectively), overall, as well as within cases of single carcinogenic HPV infections (OR: 6.6 and 9.0, respectively) and cases with presence of multiple carcinogenic infections (OR: 5.3 and 6.5, respectively). While other carcinogenic HPV genotypes had higher point estimates of Odds ratios than HPV16, their relative rarity led to wide 95% confidence intervals. When present as single carcinogenic HPV infections, HPV16, HPV31 and HPV33 had statistically significant higher risk of being present in CIN2+ while HPV16 and HPV33 had higher risk of CIN3. In cases with multiple carcinogenic infections, HPV16, HPV18, HPV56 and HPV58 had higher risk of being present in CIN2+ lesions while HPV58 and HPV68 had higher risk of being present in CIN3 lesions. An exploratory analysis of the risks of various single/multiple combinations of carcinogenic and non/unknown carcinogenic types is presented in Table S1, although the small numbers precluded the estimation in most models.

## Discussion

In this cross sectional study, we have documented the diverse distribution of HPV genotypes and their associations with rigorously confirmed cervical precancerous disease endpoints, and factors associated with HPV infection among HIV-infected women in India. Overall, the prevalence of any HPV genotypes was 52.5% and prevalence of any ‘carcinogenic’ HPV genotypes was 35.3% in this cohort of HIV-infected women. The global estimates of HPV prevalence among HIV-infected women have varied by region and the level of the HIV epidemic. [13] Studies in HIV-infected women from countries with generalized HIV epidemics, particularly in Africa, have reported a high (45–90%) carcinogenic HPV prevalence [14], [15], [16], [17] while studies from concentrated or low-level HIV epidemics in Asia, including those from India [4], [5], [18], [19], Latin America [20], [21], Europe [22], [23], and North America [24], [25] have reported lower (20–40%) carcinogenic HPV prevalence rates among HIV-infected women. The differences in HPV prevalence in different geographical locales may be attributed to differing behavioral and immunological status of the participants, as well as the differences in primers and sensitivity of the assays used for PCR.

HPV16 was the commonest genotype (carcinogenic or otherwise) in our study in this population. It has been hypothesized HPV16 has better evolutionary ability to escape the effects of immune surveillance, while non-HPV16 genotypes are often better controlled by immune response. [26], [27] Some studies, especially in those conducted in severely immunocompromised women and those not accessing ART [14], [27], [28] have reported higher relative preponderance of non-HPV16 genotypes. This is likely reflective of the loss of immune control against non-HPV16 genotypes in the context of severe immune suppression, and thus a relative preponderance along with HPV16. [14], [26] Yet, in our study we did not observe an increase of non-HPV16 carcinogenic genotypes with worsening immune status; while HPV16 was higher in women with lower CD4 counts and those currently taking cART. In fact, other than HPV16, we did not see any differences by ART status in any carcinogenicity grouping (data not shown). However, we did not have data on duration of ART to further explore differences between immune-replete and immunocompetent women on ART.

The overall diversity of HPV genotypes (regardless of carcinogenic grouping) found in our study is a characteristic uniformly reported from HIV-infected populations worldwide. This is in contrast to the substantially less diversity noted among HIV-uninfected women. [13], [28] The etiologic significance of this diversity and multiplicity, particularly those of concurrent ‘non/unknown carcinogenic’ HPV genotypes, often detected in the context of HIV-related immunosuppression, is not well understood. [12], [29], [30], [31] That said, our results suggest that future broad spectrum (polyvalent) HPV vaccines may have better efficacy in preventing CIN in higher risk groups like HIV-infected women, than the currently available bivalent (HPV16/18) or quadrivalent (HPV6/11/16/18) vaccines. [32].

Over half of our participants with HPV infection (74/146, 50.7%) had prevalent detection of multiple (≥2) HPV genotypes. The prevalence of multiple genotypes has varied widely (between 12%–87%) in similar studies worldwide [17], [33], [34], [35], [36], and has been associated with decreased immune response leading to reactivation of latent HPV genotypes, or reflective of high-risk sexual behaviors of HIV-infected women or their partners. Both explanations appear likely in our study, given the increasing multiplicity of HPV genotypes in women with lower immune status and higher risk behavior (reflected by number of lifetime sexual partners). Since multiplicity of HPV infections confounds exact genotype-specific attribution in cervical lesions, we explored the risk of cervical high grade neoplastic lesions (CIN3 and CIN2+) at an individual genotype-specific level, by stratifying as single or multiple carcinogenic genotypes (Table 3). While often limited by the small samples size, we have provided a framework for analysis that can be replicated in larger studies (with more CIN3/ICC endpoints) to evaluate individual genotype-specific attributions and elucidate the etiologic role of multiple infections in cervical carcinogenesis. [37], [38], [39].

**Table 3:** Relationship of prevalent carcinogenic HPV genotypes (present singly or concurrently with carcinogenic types) with risk of CIN2+ and CIN3 in HIV-infected women in Pune, India.

	Risk of CIN2+(vs. <CIN1)			Risk of CIN3 (vs. <CIN2)		
	Presence of anyHPV type	Presence of single carcinogenic HPV type	Presence of multiple (>2) carcinogenic HPV types	Presence of anyHPV type	Presence of single carcinogenic HPV type	Presence of multiple (>2) carcinogenic HPV types
	*OR (95%CI)* 1	*OR (95%CI)* 1	*OR (95%CI)* 2	*OR (95%CI)* 3	*OR (95%CI)* 3	*OR (95%CI)* 3
HPV16	6.7 (2.4, 18.8)	9.0 (2.8,28.7)	6.5 (1.2, 37.2)	5.2(1.6, 17.1)	6.6 (1.8, 24.4)	5.3 (0.5, 52.4)
HPV18	7.6 (1.1, 51.0)	–	8.9 (1.6, 48.9)	2.1 (0.2, 17.8)	–	6.9 (0.7, 69.9)
HPV31	6.7 (1.1, 40.3)	8.2 (1.3, 52.8)	–	2.6 (0.3, 22.9)	3.9 (0.4, 35.1)	–
HPV33	26.0 (3.4, 198.9)	23.0 (2.6, 202.0)	–	9.3 (1.6,53.6)	26 (3.3, 206.9)	–
HPV35	0.9 (0.1, 6.8)	6.6 (0.6, 76.3)	2.2 (0.2, 21.9)	–	–	–
HPV39	1.8 (0.2 19.0)	3.6 (0.4, 34.6)	–	2.1 (0.2, 17.8)	2.9 (0.3, 25.2)	–
HPV45	7.8 (0.4, 148.8)	–	9.4 (0.5, 173.9)	–	–	–
HPV51	0.3 (0.0, 3.9)	–	6.9(0.7, 68.1)	–	–	–
HPV52	–	–	–	–	–	–
HPV56	2.8 (0.6, 14.6)	3.4 (0.4, 32.9)	7.9 (1.2, 55.1)	–	–	–
HPV58	7.3 (0.8, 66.1)	–	12.4 (1.5, 99.6)	4.3 (0.5, 39.6)	–	12.3 (1.1, 135.6)
HPV59	0.4 (0, 7.6)	–	2.8 (0.3, 31.0)	–	–	–
HPV68	1.6 (0.1, 21.3)	–	3.2 (0.3, 34.0)	5.4 (0.6, 51.8)	–	16.4 (1.4, 194.1)

Footnotes to Table 3: Abbreviations: OR: Odds ratios, 95%CI: Lower limits and upper limits of the 95% Confidence intervals, CIN: Cervical intraepithelial neoplasia, HPV: human papillomavirus.

1 Odds ratios adjusted for age, number of lifetime sexual partners, CD4+ cell counts, and presence of other carcinogenic HPV types.

2 Odds ratios adjusted for age, number of lifetime sexual partners, and CD4+ cell counts.

3 Odds ratios not adjusted for any factors due to small sample size.

We found that ≥2 lifetime sexual partners are associated with presence of any HPV infection, carcinogenic HPV infection, presence of multiple carcinogenic HPV types, and non-HPV16 carcinogenic types, but not with presence of single carcinogenic types, and with HPV16. This might be explained by the fact that HPV16 (which was also the commonest carcinogenic type present singly) is highly transmittable in comparison with other carcinogenic types, thus its prevalent detection is regardless of the multiplicity of sexual partners. It was also noteworthy that other bio-behavioral factors (e.g., parity, tobacco use) which are significant HPV co-factors in cervical carcinogenesis were not significantly associated with HPV infection status. However, we lacked adequate power to study these associations with HPV among women with high-grade cervical disease status. Elucidation of the independent or combined role of such cofactors affecting risk of carcinogenic HPV and incident cervical precancer and cancer will only be studied in prospective study designs.

With the advantages of simultaneous detection of multiple genotypes, the LA-HPV assay also has certain limitations like cross-hybridization of the HPV52 probe with that of HPV33, HPV35, and HPV58 (although we only analyzed HPV52 without concurrent present of these other genotypes), lack of quantitation of HPV viral load, and chances of carry-over contamination. [40] Yet, it remains the most widely used and comprehensive commercially available assay for detection of a wide range of HPV genotypes simultaneously.

India has a high case burden of HIV/AIDS (estimated 2.4 million persons, including 1 million women) as well as a high incidence of ICC (estimated 130,000 new cases and 74,000 deaths annually). [41], [42] HIV-infected women in India are now living longer due to improved access to affordable cART in recent years. In absence of effective cervical cancer prevention services, HIV-infected women are at increased risk of ICC. Our findings add to the national and global data of HPV genotypes among HIV-infected populations. This evidence could inform the projected effectiveness of prophylactic vaccination strategies, provide background data for cost effectiveness and decision analysis models, and inform the design of HPV-based genotyping assays and biomarkers as improved screening strategies. [43] The high prevalence of carcinogenic HPV reinforces the importance of regular and vigilant screening for cervical cancer and anogenital tract pathologies in this population, especially among those with lower CD4+ counts. The results also emphasize the need for larger and prospective cohort studies to further elucidate the association between immunosuppression and HPV risk, and the etiologic significance of multiple HPV infections among HIV-infected women.

## Supporting Information

Table S1 Relationship of prevalent carcinogenic and concurrent non/unknown-carcinogenic types (single or multiple) with risk of CIN2+ and CIN3 in HIV-infected women.
(DOC)

## References

[1] Sahasrabuddhe VV, Bhosale RA, Joshi SN, Kavatkar AN, Nagwanshi CA (2010). Prevalence and predictors of colposcopic-histopathologically confirmed cervical intraepithelial neoplasia in HIV-infected women in India.. PLoS One.

[2] Strickler HD, Burk RD, Fazzari M, Anastos K, Minkoff H (2005). Natural history and possible reactivation of human papillomavirus in human immunodeficiency virus-positive women.. J Natl Cancer Inst.

[3] Palefsky JM, Holly EA (2003). Chapter 6: Immunosuppression and co-infection with HIV.. J Natl Cancer Inst Monogr.

[4] Peedicayil A, Thiyagarajan K, Gnanamony M, Pulimood SA, Jeyaseelan V (2009). Prevalence and risk factors for human papillomavirus and cervical intraepithelial neoplasia among HIV-positive women at a tertiary level hospital in India.. J Low Genit Tract Dis.

[5] Sarkar K, Pal R, Bal B, Saha B, Bhattacharya S (2011). Oncogenic HPV among HIV infected female population in West Bengal, India.. BMC Infect Dis.

[6] Joshi SN, Gopalkrishna V, Kumar BK, Dutta S, Nyaynirgune P (2005). Cervical squamous intra-epithelial changes and human papillomavirus infection in women infected with human immunodeficiency virus in Pune, India.. J Med Virol.

[7] Solomon D, Davey D, Kurman R, Moriarty A, O'Connor D (2002). The 2001 Bethesda System: terminology for reporting results of cervical cytology.. JAMA.

[8] Richart RM (1990). A modified terminology for cervical intraepithelial neoplasia.. Obstet Gynecol.

[9] Coutlee F, Gravitt P, Kornegay J, Hankins C, Richardson H (2002). Use of PGMY primers in L1 consensus PCR improves detection of human papillomavirus DNA in genital samples.. J Clin Microbiol.

[10] Coutlee F, Rouleau D, Petignat P, Ghattas G, Kornegay JR (2006). Enhanced detection and typing of human papillomavirus (HPV) DNA in anogenital samples with PGMY primers and the Linear array HPV genotyping test.. J Clin Microbiol.

[11] Kornegay JR, Roger M, Davies PO, Shepard AP, Guerrero NA (2003). International proficiency study of a consensus L1 PCR assay for the detection and typing of human papillomavirus DNA: evaluation of accuracy and intralaboratory and interlaboratory agreement.. J Clin Microbiol.

[12] Bouvard V, Baan R, Straif K, Grosse Y, Secretan B (2009). A review of human carcinogens–Part B: biological agents.. Lancet Oncol.

[13] Clifford GM, Goncalves MA, Franceschi S (2006). Human papillomavirus types among women infected with HIV: a meta-analysis.. AIDS.

[14] Sahasrabuddhe VV, Mwanahamuntu MH, Vermund SH, Huh WK, Lyon MD (2007). Prevalence and distribution of HPV genotypes among HIV-infected women in Zambia.. Br J Cancer.

[15] Singh DK, Anastos K, Hoover DR, Burk RD, Shi Q (2009). Human papillomavirus infection and cervical cytology in HIV-infected and HIV-uninfected Rwandan women.. J Infect Dis.

[16] Moodley JR, Constant D, Hoffman M, Salimo A, Allan B (2009). Human papillomavirus prevalence, viral load and pre-cancerous lesions of the cervix in women initiating highly active antiretroviral therapy in South Africa: a cross-sectional study.. BMC Cancer.

[17] Didelot-Rousseau MN, Nagot N, Costes-Martineau V, Valles X, Ouedraogo A (2006). Human papillomavirus genotype distribution and cervical squamous intraepithelial lesions among high-risk women with and without HIV-1 infection in Burkina Faso.. Br J Cancer.

[18] Bollen LJ, Chuachoowong R, Kilmarx PH, Mock PA, Culnane M (2006). Human papillomavirus (HPV) detection among human immunodeficiency virus-infected pregnant Thai women: implications for future HPV immunization.. Sex Transm Dis.

[19] Hernandez BY, Vu Nguyen T (2008). Cervical human papillomavirus infection among female sex workers in southern Vietnam.. Infect Agent Cancer.

[20] Melgaco FG, Rosa ML, Augusto EF, Haimuri JG, Jacintho C (2011). Human papillomavirus genotypes distribution in cervical samples from women living with human immunodeficiency virus.. Arch Gynecol Obstet.

[21] Coelho Lima BM, Golub JE, Tonani Mattos A, Freitas LB, Cruz Spano L (2009). Human papillomavirus in women with and without HIV-1 infection attending an STI clinic in Vitoria, Brazil.. J Int Assoc Physicians AIDS Care (Chic).

[22] Tornesello ML, Duraturo ML, Giorgi-Rossi P, Sansone M, Piccoli R (2008). Human papillomavirus (HPV) genotypes and HPV16 variants in human immunodeficiency virus-positive Italian women.. J Gen Virol.

[23] Kitchener H, Nelson L, Adams J, Mesher D, Sasieni P (2007). Colposcopy is not necessary to assess the risk to the cervix in HIV-positive women: an international cohort study of cervical pathology in HIV-1 positive women.. Int J Cancer.

[24] Hankins C, Coutlee F, Lapointe N, Simard P, Tran T (1999). Prevalence of risk factors associated with human papillomavirus infection in women living with HIV. Canadian Women's HIV Study Group.. CMAJ.

[25] Luque AE, Jabeen M, Messing S, Lane CA, Demeter LM (2006). Prevalence of human papillomavirus genotypes and related abnormalities of cervical cytological results among HIV-1-infected women in Rochester, New York.. J Infect Dis.

[26] Strickler HD, Palefsky JM, Burk RD (2008). HPV types present in invasive cervical cancers of HIV-seropositive women.. Int J Cancer.

[27] Strickler HD, Palefsky JM, Shah KV, Anastos K, Klein RS (2003). Human papillomavirus type 16 and immune status in human immunodeficiency virus-seropositive women.. J Natl Cancer Inst.

[28] McKenzie ND, Kobetz EN, Hnatyszyn J, Twiggs LB, Lucci JA 3rd (2010). Women with HIV are more commonly infected with non-16 and −18 high-risk HPV types.. Gynecol Oncol.

[29] Broker TR, Jin G, Croom-Rivers A, Bragg SM, Richardson M (2001). Viral latency–the papillomavirus model.. Dev Biol (Basel) 106: 443–451; discussion 452–443, 465–475.

[30] Palefsky JM, Minkoff H, Kalish LA, Levine A, Sacks HS (1999). Cervicovaginal human papillomavirus infection in human immunodeficiency virus-1 (HIV)-positive and high-risk HIV-negative women.. J Natl Cancer Inst.

[31] Nicol AF, Nuovo GJ, Salomao-Estevez A, Grinsztejn B, Tristao A (2008). Immune factors involved in the cervical immune response in the HIV/HPV co-infection.. J Clin Pathol.

[32] Stanley M (2010). Prospects for new human papillomavirus vaccines.. Curr Opin Infect Dis.

[33] Levi JE, Kleter B, Quint WG, Fink MC, Canto CL (2002). High prevalence of human papillomavirus (HPV) infections and high frequency of multiple HPV genotypes in human immunodeficiency virus-infected women in Brazil.. J Clin Microbiol.

[34] Luque AE, Hitti J, Mwachari C, Lane C, Messing S (2010). Prevalence of human papillomavirus genotypes in HIV-1-infected women in Seattle, USA and Nairobi, Kenya: results from the Women's HIV Interdisciplinary Network (WHIN).. Int J Infect Dis.

[35] Hawes SE, Critchlow CW, Sow PS, Toure P, N'Doye I (2006). Incident high-grade squamous intraepithelial lesions in Senegalese women with and without human immunodeficiency virus type 1 (HIV-1) and HIV-2.. J Natl Cancer Inst.

[36] Moscicki AB, Ellenberg JH, Farhat S, Xu J (2004). Persistence of human papillomavirus infection in HIV-infected and -uninfected adolescent girls: risk factors and differences, by phylogenetic type.. J Infect Dis.

[37] Nuovo GJ, Darfler MM, Impraim CC, Bromley SE (1991). Occurrence of multiple types of human papillomavirus in genital tract lesions. Analysis by in situ hybridization and the polymerase chain reaction.. Am J Pathol.

[38] Vaccarella S, Franceschi S, Snijders PJ, Herrero R, Meijer CJ (2010). Concurrent infection with multiple human papillomavirus types: pooled analysis of the IARC HPV Prevalence Surveys.. Cancer Epidemiol Biomarkers Prev.

[39] Wentzensen N, Schiffman M, Dunn T, Zuna RE, Gold MA (2009). Multiple human papillomavirus genotype infections in cervical cancer progression in the study to understand cervical cancer early endpoints and determinants.. Int J Cancer.

[40] Steinau M, Swan DC, Unger ER (2008). Type-specific reproducibility of the Roche linear array HPV genotyping test.. J Clin Virol.

[41] Joint United Nations Programme on HIV/AIDS (2010). Global report: UNAIDS report on the global AIDS epidemic. Geneva, Switzerland: Joint United Nations Programme on HIV/AIDS. pp.. v.

[42] Ferlay J, Shin HR, Bray F, Forman D, Mathers C (2010). Estimates of worldwide burden of cancer in 2008: GLOBOCAN 2008.. Int J Cancer.

[43] Sahasrabuddhe VV, Luhn P, Wentzensen N (2011). Human papillomavirus and cervical cancer: biomarkers for improved prevention efforts.. Future Microbiol.

