# Supplementary material for: HPV Genotype Distribution in Cervical Intraepithelial Neoplasia among HIV-Infected Women in Pune, India
Source: PLoS One. 2012 Jun 19;7(6):e38731. doi: 10.1371/journal.pone.0038731 (PMC3378535; doi:10.1371/journal.pone.0038731)
Supplement: Table S1 — Relationship of prevalent carcinogenic and concurrent non/unknown-carcinogenic types (single or multiple) with risk of CIN2+ and CIN3 in HIV-infected women. (DOC) [file pone.0038731.s001.doc]

**Supporting Table 1:** Relationship of prevalent carcinogenic and concurrent non/unknown-carcinogenic types (single or multiple) with risk of CIN2+ and CIN3 in HIV-infected women in Pune, India.

|  | **Risk of CIN2+(vs. <CIN1)** | | | | **Risk of CIN3 (vs. <CIN2)** | | | |
| --- | --- | --- | --- | --- | --- | --- | --- | --- |
|  | Presence of single carcinogenic HPV type  and  single non-carcinogenic HPV type | Presence of single carcinogenic HPV type  and  multiple non-carcinogenic HPV types | Presence of multiple carcinogenic HPV types  and  single non-carcinogenic HPV type | Presence of multiple carcinogenic HPV types  and  multiple non-carcinogenic HPV types | Presence of single carcinogenic HPV type  and  single non-carcinogenic HPV type | Presence of single carcinogenic HPV type  and  multiple non-carcinogenic HPV types | Presence of multiple carcinogenic HPV types  and  single non-carcinogenic HPV type | Presence of multiple carcinogenic HPV types  and  multiple non-carcinogenic HPV types |
|  | *OR (95%CI)** | *OR (95%CI)* | *OR (95%CI)* | *OR (95%CI)* | *OR (95%CI)* | *OR (95%CI)* | *OR (95%CI)* | *OR (95%CI)* |
|  |  |  |  |  |  |  |  |  |
| HPV16 | 2.7 (0.2, 25.3) | - | 31.6 (2.6,381.8) | 5.9 (0.5, 63.0) | - | - | 37.3 (2.3,598.3) | - |
| HPV18 | - | - | 15.7 (1.9,124.5) | - | - | - | - | - |
| HPV31 | - | - | - | - | - | - | - | - |
| HPV33 | 30.6 (2.6,366.9) | - | - | - | 161(10, 2585.1) | - | - | - |
| HPV35 | - | - | 4.7 (0.4, 49.0) | - | - | - | - | - |
| HPV39 | 3.4 (0.4, 33.2) | - | - | - | - | - | - | - |
| HPV45 | - | - | - | - | - | - | - | - |
| HPV51 | - | - | - | 5.9 (0.5, 63.0) | - | - | - | - |
| HPV52 | - | - | - | - | - | - | - | - |
| HPV56 | 6.9 (0.6, 82.3) | - | 14.2 (0.8,244.3) | 17.9 (1.0,312.7) | - | - | - | - |
| HPV58 | - | - | 7.1 (0.6, 84.6) | 17.9 (1.0,312.7) | - | - | 37.3 (2.3,598.3) | - |
| HPV59 | - | - | 14.2 (0.8,244.3) | - | - | - | - | - |
| HPV68 | - | - | - | - | - | - | - | - |

Footnotes to Supplementary Table 1: Abbreviations: OR: Odds ratios, 95%CI: Lower limits and upper limits of the 95% Confidence intervals, CIN: Cervical intraepithelial neoplasia, HPV: human papillomavirus.

Odds ratios presented in this table were not adjusted for any factors due to small sample sizes.
